# Clinical application of a novel high-selectivity steerable-tip catheter for endoscopic retrograde cholangiopancreatography in patients with altered surgical anatomy

**DOI:** 10.1055/a-2362-0666

**Published:** 2024-07-29

**Authors:** Osamu Inatomi, Atsushi Yamada, Shuhei Shintani, Kosuke Hiroe, Hidenori Kimura, Atsushi Nishida, Tohru Tani

**Affiliations:** 1 13051 Division of Gastroenterology, Department of Medicine, Shiga University of Medical Science, Otsu, Japan; 2 13051 Medical Innovation Research Center, Shiga University of Medical Science, Otsu, Japan; 3 13051 Department of Endoscopy, Shiga University of Medical Science, Otsu, Japan; 4 13051 Department of Advanced Medical Research and Development, Shiga University of Medical Science, Otsu, Japan

Endoscopic retrograde cholangiopancreatography (ERCP) for the selective treatment of the biliary branch is frequently challenging in patients with surgically altered intestinal tracts 1 2 3 . We have developed and commercialized a novel steerable catheter for ERCP (KC226; Zeon Medical, Tokyo, Japan) capable of balanced bidirectional tip-bending at steep angles by using a seamless tube with a distal part made of an artificial blood vessel material and a wire-driven antagonistic mechanism 4 ( Fig. 1 ). This study evaluates the efficacy and safety of this catheter inserted into balloon enteroscopes for accessing the biliary tract in patients with surgically altered anatomy.

**Fig. 1:**
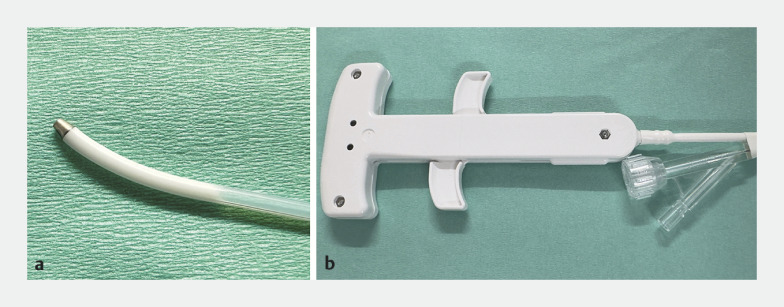
Image of the novel tip-steerable catheter. This catheter measures 2200 mm in length with an outer diameter of 2.1 mm. The tip can bend bidirectionally up to 90°, controlled by pulling two wires via a controller attached at the base. The insertion port is designed for 0.035-inch guidewires and is the injection site for contrast agents.

We compared its endoscopic bending performance with a conventional steerable-tip catheter (PR-233Q; Olympus Medical Systems, Tokyo, Japan) through desktop experiments using a balloon enteroscope. Results demonstrated that the novel catheter could achieve bidirectional tip bend angles of ± 90° within a 10-mm radius, significantly enhancing maneuverability ( Video 1 ).

This video demonstrates tip-steerable cathetersʼ trackability in bile duct-seeking during endoscopic retrograde cholangiopancreatography procedures performed with a balloon enteroscope alongside a comparative experiment on the bending performance.Video 1

Clinically, we evaluated four consecutive patients (mean age 74 years) who had undergone Roux-en-Y cholangiojejunostomy, presenting symptoms such as acute cholangitis and recurrent abdominal pain. With the conventional steerable-tip catheter, guidewire (GW) insertions in all cases were limited to the bile duct of a single lobe, and it was impossible to perform cholangiography across bilateral lobes. However, switching to the newly developed catheter in all cases could complete bilateral bile duct cannulation, GW insertions ( Video 1 , Fig. 2 ), and cholangiography successfully and swiftly under single- or double-balloon enteroscopy in 2.8 minutes on average. No catheter-related complications were observed.

**Fig. 2:**
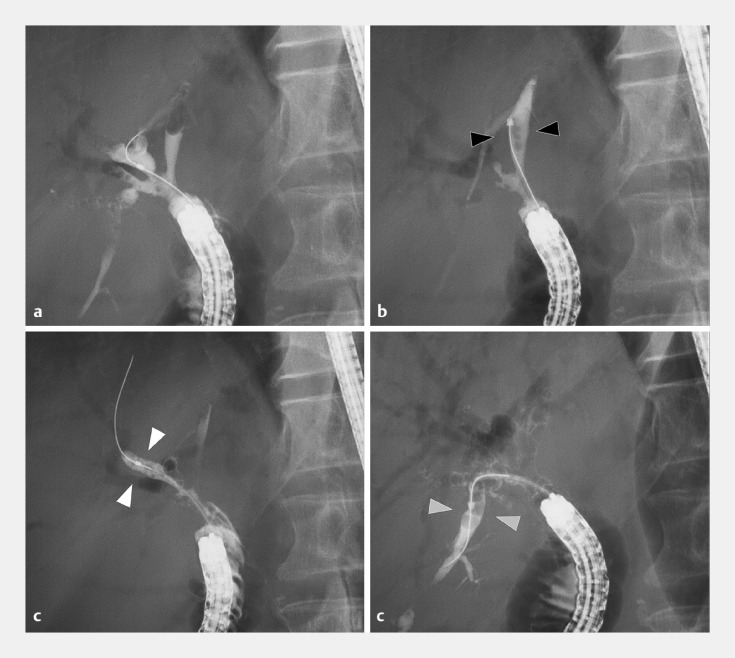
Cholangiography image from a clinical case. A 70-year-old man with surgically altered intestinal tracts due to pancreaticoduodenectomy experienced repeated episodes of acute cholangitis. **a** Cholangiography revealed multiple stenoses in the right bile duct, attributed to recurrent inflammation. **b** The tip-steerable catheter efficiently completed guidewire insertion into the left bile duct. **c** Insertion into the anterior branches. **d** Insertion into the right bile duct.

The catheter-tip steerability proved particularly beneficial in navigating the complex biliary anatomy post-cholangiojejunostomy, effectively overcoming the typical challenges posed by altered anastomotic angles between the bile duct and jejunum. Thus, this novel catheter may be valuable for challenging bile duct access in patients with surgically reconstructed intestinal tracts.

Endoscopy_UCTN_Code_TTT_1AR_2AZ

## Notes

Endoscopy E-Videos https://eref.thieme.de/e-videos
*E-Videos* is an open access online section of the journal *Endoscopy* , reporting on interesting cases and new techniques in gastroenterological endoscopy. All papers include a high-quality video and are published with a Creative Commons CC-BY license. Endoscopy E-Videos qualify for HINARI discounts and waivers and eligibility is automatically checked during the submission process. We grant 100% waivers to articles whose corresponding authors are based in Group A countries and 50% waivers to those who are based in Group B countries as classified by Research4Life (see: https://www.research4life.org/access/eligibility/ ). This section has its own submission website at https://mc.manuscriptcentral.com/e-videos .
